# Supplementary material for: Detection and Monitoring of Tumor-Derived Mutations in Circulating Tumor DNA Using the UltraSEEK Lung Panel on the MassARRAY System in Metastatic Non-Small Cell Lung Cancer Patients
Source: Int J Mol Sci. 2023 Aug 29;24(17):13390. doi: 10.3390/ijms241713390 (PMC10487510; doi:10.3390/ijms241713390)
Supplement: Supplementary file 1 [file ijms-24-13390-s001.zip › ijms-2558089-supplementary.pdf]

Supplemental Material

Detection and monitoring of tumor-derived mutations in circulating tumor DNA using the UltraSEEK Lung Panel on the MassARRAY System in metastatic non-small cell lung cancer patients

Table of content

Supplemental Materials and Methods 2

Sample processing 2

CcfDNA extraction and quantification 2

Molecular analysis using the UltraSEEK® Lung Panel on the MassARRAY® System 2

Molecular analysis using ddPCR 3

Supplemental Tables 5

Supplemental Table 1. Mutations detectable on the UltraSEEK® Lung Panel and applied ddPCR assays 5

Supplemental Table 2. Elaborate mutation detection of Seraseq® reference material using the UltraSEEK® Lung Panel. 7

Supplemental Table 3. Agreement of UltraSEEK® and ddPCR in the detection of mutations in plasma. 8

Supplemental Table 4. Detection of tumor tissue-derived variants in the baseline plasma sample using the UltraSEEK® Lung Panel. 12

Supplemental Table 5. Validation of undetected tumor-derived variants in baseline plasma using UltraSEEK® with mutation-specific ddPCR. 14

Supplemental Table 6. Stepwise Cox regression analysis depicting the discriminators for PFS and OS 15

Supplemental Table 7. Comparison of ctDNA dynamics between UltraSEEK® and ddPCR. 16

Supplemental Table 8. Detection of acquired resistance mutations using the UltraSEEK® Lung Panel. 19

Supplemental Table 9. Primer and probe sequences of the custom ddPCR assays. 20

Supplemental Figures 21

Supplemental Figure 1. Concordance of ctDNA dynamics between UltraSEEK® and ddPCR 21

Supplemental References 22

Supplemental Materials and Methods

Sample processing

Plasma samples were collected in either EDTA blood collection tubes (BCTs; Becton Dickinson, Franklin Lakes, NJ, USA), Cell-Free DNA BCTs® (Streck, Omaha, NE, USA) or PAXgene Blood DNA Tubes (Qiagen, Hilden, Germany). Blood samples were processed within 4 hours (EDTA and PAXgene) or 24 hours (Streck). EDTA samples were centrifuged at 820×*g* (University Medical Center Groningen; UMCG) or 200×*g* (University Medical Center Hamburg-Eppendorf; UKE) for 10 minutes to separate lymphocytes from plasma; other blood samples were centrifuged at 1,600×*g* for 10 minutes (Streck) or 1,900×*g* for 15 minutes (PAXgene). The supernatant was subsequently centrifuged for 10 minutes at 16,000×*g* to pellet the remaining debris. Cell-free plasma was stored in 1mL fractions at -80°C until ccfDNA extraction. All patients provided written informed consent.

CcfDNA extraction and quantification

Circulating cell-free DNA (ccfDNA) was extracted from 2mL (range 1.5-4mL) of blood plasma and eluted in 30 or 52µL AVE elution buffer with QiaAMP Circulating Nucleic Acid Kit (Qiagen) according to the manufacturer's recommendations [1–3]. Single-reaction assessment of multiple preanalytical parameters was performed using 1.5µL extracted ccfDNA from each sample with the LiquidIQ® Panel with matrix assisted laser desorption ionization time-of-flight (MALDI-TOF)-based analysis on the MassARRAY® System (Agena Bioscience, San Diego, CA, USA) as previously described [4]. Following the standard operating procedures for ddPCR analysis of the UMCG, ccfDNA yield and droplet digital PCR (ddPCR) input were determined with the Qubit 1x dsDNA HS Assay Kit (Thermofisher Scientific, Waltham, MA, USA), which correlated strongly (r^2=0.87, P<0.0001) with previous LiquidIQ® analysis [4].

Molecular analysis using the UltraSEEK® Lung Panel on the MassARRAY® System

CcfDNA was analyzed on the MassARRAY® System using the UltraSEEK® Lung Panel (Agena Bioscience) that covers 73 hotspot mutations across five genes relevant to NSCLC (*BRAF*, *EGFR*, *ERBB2*, *KRAS* and *PIK3CA*; Supplemental Table 1). The UltraSEEK® workflow [5–9] was conducted in all three institutions (UMCG, UKE and Institut d'Analyse Génomique Imagenome, Montpellier) according to manufacturer's instructions. The assay consists of a single multiplex PCR reaction targeting specific regions of the five genes, followed by a single base extension relative to the specific mutation using chain terminators. In detail, single multiplexed PCR allows up to 35µL sample in a total reaction volume of 70µL. An optimal ccfDNA input of 10ng for the UltraSEEK® Lung Panel compared to Cobas® EGFR Mutation Test v2 (100% concordance) and a lower concordance of 83% using less ccfDNA input was reported recently [4]. Optimally, for each sample, a ccfDNA input of 10ng was used for each sample, except when ccfDNA concentrations were too low. Reactions were incubated initially at 95°C for 2 minutes. Forty-five cycles of PCR were performed at 95°C for 30 seconds, 56°C for 30 seconds, and 72°C for 1 minute. The PCR was completed with a final incubation of 5 minutes at 72°C. Amplified products were treated with shrimp alkaline phosphatase for 40 minutes at 37°C, followed by denaturation for 5 minutes at 85°C. Single-base extension with biotinylated chain terminating nucleotides is then incorporated only when the mutant allele is present allowing for further enrichment of the mutant signal. Single-base extension with biotinylated chain terminator nucleotides specific to the mutant allele was performed at 95°C for 30 seconds, followed by 40 cycles at 94°C for 5 seconds with five nested cycles of 52°C for 5 seconds and 80°C for 5 seconds and a final incubation at 72°C for 3 minutes. Streptavidin-coated magnetic beads were used to capture the single-base extended oligonucleotides. Beads with captured products were pelleted using a magnet, suspended with 13µL of biotin competition solution, and then incubated at 90°C for 5 minutes.

The final eluted enriched extension products were transferred into the MassARRAY® System for automated sample handling including desalting with anion exchange resin, dispensing analytes onto the SpectroCHIP® Array (Agena Bioscience) and data acquisition via MALDI-TOF mass spectrometry as previously described [7]. Primary data analysis was performed using MassARRAY® Typer software version 5.0.5 (Agena Bioscience). Variant allele calls and frequencies (VAF) were returned by the automated Somatic Variant Report (SVR) software version 1.0.5 specific for the UltraSEEK® Lung Panel and allele calls with a signal-to-noise ratio of seven and a z-score of seven were considered

significant. For allele calling, the reporter algorithm takes an instrument specific baseline for each mutation assay into account. Herein, the assay specific noise is assessed by analyzing a cohort of wild-type samples and mutant call significance was controlled by analyzing commercial mutations controls as a titration of mutant allele frequencies down to the limit of detection (LoD) of 0.1%. VAF calculation within SVR is based on the normalized peak intensity of the mutant allele and an assay-specific correction coefficient. The mutant peak intensity is normalized against the linear regression curve for the five capture control peaks found in the spectrum. The capture control peaks are biotin-labeled, nonreactive oligos, which are added to the extension reaction and used as an internal control for the streptavidin-bead capture and elution of the mutant extension product steps. The assay-specific correction coefficients derived from titration experiments down from the LoD (in most cases 0.125%, see Supplemental Table 1) to 2% conducted for each variant covered by the UltraSEEK® Lung Panel using synthetic positive controls. Titration curves were embedded in SVR to semiquantitatively estimate variant allele frequencies (VAFs; also see https://www.agenabio.com/wp-content/uploads/2022/08/Agena-Bioscience-USK-Lung-WhitePaper-ONC003101.pdf).

The UltraSEEK® Lung Panel on the MassARRAY® System was recently validated in a multicenter ring trial using the commercially available Seraseq® ctDNA Complete™ Mutation Mix (Seracare Life Sciences, Milford, MA, USA) reference material, which contains ten clinically relevant mutations at 1% VAF detectable with the UltraSEEK® Lung Panel, and compared with different next generation sequencing (NGS)-based and ddPCR assays [8]. In the present study, to assess the interlaboratory analytical sensitivity and specificity, as well as the false-positive and false-negative rates of the UltraSEEK® Lung Panel, a multicenter interlaboratory study using the same reference material was performed. Samples containing different mutations with low VAFs (0.1%, 0.5%, 1.0% and 2.5%) and different DNA input amounts (5, 10 and 20ng) were analyzed in each laboratory. To verify reproducibility between runs and laboratories, Seraseq® ctDNA Complete™ Mutation Mix at 0.5% VAF and Seraseq® ctDNA Complete™ Wildtype were included as an internal control on each plate. An overall detection rate of 87.2% was observed (Supplemental Table 2). Regarding quantification, an average inter-run coefficient of variance across all variants determined in the internal control samples on all plates of 36% was determined. The intra-run coefficient of variance based on the multicenter interlaboratory study using the same reference material was 14.8%. Since all samples from each patient were analyzed on the same plate, with respect to dynamics of ctDNA levels, only changes in VAF equal to or greater than 15% between start of treatment (t_0) and first treatment evaluation (t_1) samples were considered an increase or decrease, as this variance is similar with the previously determined intra-run variability of UltraSEEK® analyses (10%) [8] and confirmed in stepwise cox regression analysis versus other proxies (Supplemental Table 6). In cases with two or more variants and contradictory ctDNA dynamics (referred to as mixed dynamics; *n*=3), the variant or sum of variants with the highest VAF was considered in the grouped analysis.

Molecular analysis using ddPCR

For the UMCG cohort, ddPCR analysis was performed to confirm mutant genotyping and quantification on all samples with mutations detected in plasma with the UltraSEEK® Lung Panel of that same patient. Besides, when a mutation detected in tissue was not retrieved with UltraSEEK® at baseline (t_0), ddPCR analysis was performed on that baseline and follow-up samples as well. DdPCR assays were either wet-lab validated commercially available (Bio-Rad Laboratories, Pleasanton, CA, USA; Supplemental Table 1), or custom designed using PrimerQuest and ordered from Integrated DNA Technologies (IDT, Coralville, IA, USA; Supplemental Table 9). In the UMCG, the Department of Pathology is a NEN-EN-ISO15189 accredited laboratory (Dutch Council of Accreditation registration number M257). *KRAS*, *BRAF* V600E, and custom developed *EGFR* ddPCR assays were validated according to this accreditation standard, including the assessment of assay performance, clinical and analytical sensitivity and specificity, reproducibility, and LoD using well-defined positive and negative control samples that are reflective of clinical samples. Wet-lab validated commercial ddPCR assays were verified for specificity and LoD using patient-derived clinical samples. DdPCR analyses were performed using the Bio-Rad QX200™ platform (Bio-Rad Laboratories) included positive, wildtype and no template controls as reported previously [1,3], with a PCR set-up as recommended by the manufacturer. Samples were regarded as positive if ≥3 mutant droplets were detected and negative if <3 mutant droplets with at least 330 total positive (wildtype and mutant) droplets were detected. For each ddPCR reaction, an input of

5.9ng ccfDNA was used, except when ccfDNA concentrations measured by Qubit were too low due to input volume restrictions. 5.9ng of ccfDNA theoretically equals 1770 copies and when expecting a 50% recovery efficiency (approximately 740 positive droplets), a sensitivity of <0.5% is ensured (3/740=0.4%). DdPCR data were analyzed for mutant molecule levels and VAF percentages using the QuantaSoft™ analytical software version 1.7.4.0917 and QuantaSoft™ Analysis Pro 1.0.596 (Bio-Rad). With respect to dynamics of ctDNA levels, only changes in mutant ctDNA levels greater than or equal to 31% between t_0 and t_4 were considered an increase or decrease, in accordance with the previously determined multicenter intra- and inter-run variability of ddPCR analyses [3,8] (Supplemental Table 6).

All standard precautions were taken to avoid contamination of amplification products using separate laboratories for pre- and post-PCR handling. Primer validation reports are available upon reasonable request.

Supplemental Tables

Supplemental Table S1. Mutations detectable on the UltraSEEK® Lung Panel and applied ddPCR assays.

Gene	CDS mutation	Amino acid change	COSMIC ID	Limit of detection*	DdPCR assay†
EGFR	c.2125G>A	p.(E709K)	COSM12988	0.125%	N/A
	c.2126A>C	p.(E709A)	COSM13427	0.250%	dHsaMDV2516858
	c.2126A>G	p.(E709G)	COSM13009	0.125%	N/A
	c.2126A>T	p.(E709V)	COSM12371	0.250%	N/A
	c.2155G>A	p.(G719S)	COSM6252	0.250%	dHsaEXD40054642
	c.2155G>T	p.(G719C)	COSM6253	0.125%	dHsaEXD40054642
	c.2156G>C	p.(G719A)	COSM6239	0.125%	dHsaEXD40054642
	c.2235_2246del	p.(E746_E749del)	COSM28517	0.125%	Custom drop-off assay‡
	c.2235_2249del	p.(E746_A750del)	COSM6223	0.125%	Custom drop-off assay‡
	c.2235_2248delinsAATTC	p.(E746_A750delinsIP)	COSM13550	0.125%	Custom drop-off assay‡
	c.2235_2251delinsAATTC	p.(E746_T751delinsIP)	COSM13552	0.125%	Custom drop-off assay‡
	c.2236_2250del	p.(E746_A750del)	COSM6225	0.125%	Custom drop-off assay‡
	c.2236_2253del	p.(E746_T751del)	COSM12728	0.125%	Custom drop-off assay‡
	c.2237_2251del	p.(E746_T751delinsA)	COSM12678	0.125%	Custom drop-off assay‡
	c.2237_2254del	p.(E746_S752delinsA)	COSM12367	0.125%	Custom drop-off assay‡
	c.2237_2252delinsT	p.(E746_T751delinsV)	COSM12386	0.125%	Custom drop-off assay‡
	c.2237_2253delinsTTGCT	p.(E746_T751delinsVA)	COSM12416	0.125%	Custom drop-off assay‡
	c.2237_2253delinsTTCCT	p.(E746_T751delinsVP)	COSM52935	0.125%	Custom drop-off assay‡
	c.2237_2257delinsTCT	p.(E746_P753delinsVS)	COSM18427	0.125%	Custom drop-off assay‡
	c.2237_2255delinsT	p.(E746_S752delinsV)	COSM12384	0.125%	Custom drop-off assay‡
	c.2238_2255del	p.(E746_S752delinsD)	COSM6220	0.125%	Custom drop-off assay‡
	c.2238_2248delinsGC	p.(L747_A750delinsP)	COSM12422	0.125%	Custom drop-off assay‡
	c.2239_2247del	p.(L747_E749del)	COSM6218	0.125%	Custom drop-off assay‡
	c.2239_2256del	p.(L747_S752del)	COSM6255	0.125%	Custom drop-off assay‡
	c.2239_2256delinsCAA	p.(L747_S752delinsQ)	COSM12403	0.125%	Custom drop-off assay‡
	c.2239_2248delinsC	p.(L747_A750delinsP)	COSM12382	0.125%	Custom drop-off assay‡
	c.2239_2251delinsC	p.(L747_T751delinsP)	COSM12383	0.125%	Custom drop-off assay‡
	c.2239_2258delinsCA	p.(L747_P753delinsQ)	COSM12387	0.125%	Custom drop-off assay‡
	c.2240_2251del	p.(L747_T751delinsS)	COSM6210	0.125%	Custom drop-off assay‡
	c.2240_2254del	p.(L747_T751del)	COSM12369	0.125%	Custom drop-off assay‡
	c.2240_2257del	p.(L747_P753delinsS)	COSM12370	0.125%	Custom drop-off assay‡
	c.2303G>T	p.(S768I)	COSM6241	0.250%	dHsaMDS132341354
	c.2300_2308dup	p.(A767_V769dup)	COSM12376	0.250%	N/A
	c.2309_2310delinsCCAGCGTGGAT	p.(A767_D769dup)	COSM13558	0.125%	N/A
	c.2310_2311insGGT	p.(D770_N771insG)	COSM12378	0.125%	N/A
	c.2303_2311dup	p.(S768_D770dup)	COSM13428	0.125%	N/A
	c.2311_2312insCAC	p.(N711delinsTH)	COSM22946	0.250%	N/A
	c.2311_2319dup	p.(N771_H773dup)	COSM12381	0.125%	N/A
	c.2317_2319dup	p.(H773dup)	COSM12377	0.125%	N/A
	c.2369C>T	p.(T790M)	COSM6240	0.125%	Custom assay‡
	c.2389T>A	p.(C797S)	COSM6493937	0.125%	Custom assay‡

EGFR	c.2390G>C	p.(C797S)	COSM5945664	0.125%	Custom assay‡
	c.2573T>G	p.(L858R)	COSM6224	0.250%	Custom assay‡
	c.2582T>A	p.(L861Q)	COSM6213	0.125%	N/A
	c.2582T>G	p.(L861R)	COSM12374	0.125%	N/A
BRAF	c.1406G>C	p.(G469A)	COSM460	0.125%	dHsaMDV2516932§
	c.1406G>T	p.(G469V)	COSM459	0.125%	dHsaMDS747800353§
	c.1781A>G	p.(D594G)	COSM467	0.250%	N/A
	c.1799T>A	p.(V600E)	COSM476	0.125%	dHsaMDV2010027§
ERBB2	c.2313_2324dup	p.(Y772_A775dup)	COSM20959	0.250%	N/A
	c.2314_2325dup	p.(Y772_A775dup)	COSM12558	0.125%	N/A
	c.2326_2327insTGT	p.(G776delinsVC)	COSM12553	0.125%	N/A
	c.2326_2327insTTT	p.(G776delinsVC)	COSM12552	0.125%	N/A
KRAS	c.34G>A	p.(G12S)	COSM517	0.125%	1863506
	c.34G>C	p.(G12R)	COSM518	0.125%	1863506
	c.34G>T	p.(G12C)	COSM516	0.125%	1863506
	c.34_35GG>TA	p.(G12Y)	COSM25081	0.125%	N/A
	c.35G>A	p.(G12D)	COSM521	0.125%	1863506
	c.35G>C	p.(G12A)	COSM522	0.125%	1863506
	c.35G>T	p.(G12V)	COSM520	0.125%	1863506
	c.37G>T	p.(G13C)	COSM527	0.125%	N/A
	c.38G>A	p.(G13D)	COSM532	0.125%	1863506
	c.183A>C	p.(Q61H)	COSM554	0.125%	12001626
	c.183A>T	p.(Q61H)	COSM555	0.125%	12001626
	c.181C>A	p.(Q61K)	COSM549	0.125%	12001626
	c.181C>G	p.(Q61E)	COSM550	0.125%	N/A
	c.182A>C	p.(Q61P)	COSM551	0.125%	N/A
	c.182A>G	p.(Q61R)	COSM552	0.500%	12001626
	c.182A>T	p.(Q61L)	COSM553	0.125%	12001626
PIK3CA	c.1624G>A	p.(E542K)	COSM760	0.250%	dHsaMDV2010073§
	c.1633G>A	p.(E545K)	COSM763	0.125%	dHsaMDV2010075§
	c.3140A>G	p.(H1047R)	COSM775	0.500%	N/A
	c.3140A>T	p.(H1047L)	COSM776	0.125%	N/A

*Limit of detection of UltraSEEK® assays as previously determined and published online: https://www.agenabio.com/wp-content/uploads/2022/08/Agena-Bioscience-USK-Lung-WhitePaper-ONC003101.pdf. †DdPCR assay IDs as commercially available at Bio-Rad Laboratories. ‡Custom assays were designed by Integrated DNA Technologies (IDT) and sequences are provided in Supplemental Table 9. §DdPCR assays were provided in-kind by Bio-Rad Laboratories for our NSCLC studies. All ddPCR assays were performed with a melting temperature (Tm) of 55°C and 1.1µL ready to use primer and probe mixes according to the supplier's protocol without modifications. N/A, not available.

Supplemental Table S2. Elaborate mutation detection of Seraseq® reference material using the UltraSEEK® Lung Panel.

VAF	DNA input	*BRAF* c.1799T>A p.(V600E)	*EGFR* c.2369C>T p.(T790M)	*EGFR* c.2573T>G p.(L858R)	*EGFR* c.2240_2257del p.(L747_P753delinsS)	*EGFR* c.2235_2249del p.(E746_A750del)	*ERBB2* c.2313_2324dup p.(Y772_A775dup)	*KRAS* c.34G>T p.(G12C)	*KRAS* c.35G>A p.(G12D)	*KRAS* c.183A>C p.(Q61H)	*PIK3CA* c.3140A>G p.(H1047R)	False-positive calls	Overall detection rate
	20ng	100%	100%	100%	100%	100%	100%	100%	100%	100%	100%	1	
**2.5%**	10ng	100%	100%	100%	100%	100%	100%	100%	100%	100%	100%		100.0%
	5ng	100%	100%	100%	100%	100%	100%	100%	100%	100%	100%	3	
	20ng	100%	100%	100%	100%	100%	100%	100%	100%	100%	100%		
**1.0%**	10ng	100%	100%	100%	100%	100%	100%	100%	100%	100%	100%		99.3%
	5ng	100%	100%	100%	100%	100%	100%	100%	100%	80%	100%	1	
	20ng	100%	100%	100%	86%	86%	57%	86%	100%	86%	86%		
**0.5%**	10ng	86%	100%	100%	100%	100%	57%	86%	100%	100%	100%	8*	91.0%
	5ng	100%	100%	86%	100%	100%	57%	100%	100%	86%	86%	1	
	20ng	86%	43%	14%	86%	71%	14%	14%	43%	43%	29%		
**0.1%**	10ng	100%	86%	29%	100%	86%	57%	43%	14%	57%	29%		51.4%
	5ng	57%	43%	14%	86%	86%	43%	57%	43%	43%	29%	2	
		All plates in study											
**0.5%**	10ng	100%	93%	89%	86%	89%	36%	71%	100%	86%	100%	-	87.2%

Seraseq® ctDNA Complete™ Mutation Mix at different allele frequencies was provided by SeraCare three laboratories (University Medical Center Groningen, University Medical Center Hamburg-Eppendorf and Institut d'Analyse Médicale Imagenome, Montpellier) participating in the CANCER-ID consortium. The received samples were quantified using Qubit and diluted to a concentration of 1ng/μL. Afterwards, 5, 10 or 20μL was used to achieve the required DNA input. Five replicates were analyzed for the 2.5 and 1% VAF; seven replicates were used for the 0.5 and 0.1% VAF. Twenty-two false-positive variants were identified across all 67 plates (in total 5,022 variant positions), resulting in a specificity of >99.5%. Six false-positive calls were identified in wildtype samples (one with 20ng input, two with 10ng input, and 3 with 5ng input). False-positive calls were observed across eleven samples (88% sample level specificity), of which seven had a DNA input of 5ng. Interlaboratory discordances were not attributable to performance at a single site. *All false-positives were called in the same sample. VAF, variant allele frequency.

Supplemental Table S3. Agreement of UltraSEEK® and ddPCR in the detection of mutations in plasma.

			UltraSEEK		ddPCR		
**Patient ID**	**Analyzed mutation**	**Timepoint**	**UltraSEEK input (ng)**	**VAF**	**ddPCR input (ng)**	**Mutant copies/mL plasma**	**VAF**
UMCG-AB-001	*EGFR* c.2240_2254del; p.(L747_T751del)*	t_0	3.9	0.5%	5.1	0[d]	0.0%
	EGFR c.2240_2254del; p.(L747_T751del)	t_1	7.8	0.0%	5.9	0[d]	0.0%
	EGFR c.2240_2254del; p.(L747_T751del)	t_p	10.0	0.0%	5.9	0[d]	0.0%
UMCG-AB-002	*EGFR* c.2573T>G; p.(L858R)	t_0	10.0	5.0%	5.9	673	15.3%
	EGFR c.2573T>G; p.(L858R)	t_p	9.9	5.5%	5.9	1895	12.8%
	EGFR c.2369C>T; p.(T790M)	t_0	10.0	2.9%	5.9	222	1.7%
	EGFR c.2369C>T; p.(T790M)	t_p	9.9	2.2%	5.9	777	8.0%
	EGFR c.2389T>A; p.(C797S)	t_0	10.0	0.0%	5.9	0[e]	0.0%
	EGFR c.2389T>A; p.(C797S)	t_p	9.9	4.2%	5.9	44	2.3%
UMCG-AB-003	*EGFR* c.2573T>G; p.(L858R)	t_0	5.7	3.7%	4.7	207	12.1%
	EGFR c.2573T>G; p.(L858R)	t_1	8.9	3.7%	4.5	546	17.9%
	EGFR c.2573T>G; p.(L858R)	t_p	10.0	3.4%	5.9	1055	27.9%
	EGFR c.2369C>T; p.(T790M)*	t_0	5.7	0.5%	9.2	0[c]	0.0%
	EGFR c.2369C>T; p.(T790M)	t_1	8.9	2.2%	4.5	109	4.9%
	EGFR c.2369C>T; p.(T790M)	t_p	10.0	1.9%	5.9	161	6.3%
UMCG-AB-004	*EGFR* c.2240_2254del; p.(L747_T751del)	t_0	9.9	0.4%	5.9	105	3.2%
	EGFR c.2240_2254del; p.(L747_T751del)	t_p	10.0	2.0%	5.9	596	23.0%
	EGFR c.2369C>T; p.(T790M)	t_0	9.9	0.0%	5.9	0[c]	0.0%
	EGFR c.2369C>T; p.(T790M)	t_p	10.0	2.1%	5.9	133	4.7%
UMCG-AB-006	*EGFR* c.2573T>G; p.(L858R)	t_0	6.4	2.9%	5.9	26	10.2%
	EGFR c.2573T>G; p.(L858R)	t_p	9.9	3.7%	5.9	162	36.7%
	EGFR c.2369C>T; p.(T790M)	t_0	6.4	0.4%	5.9	5	1.8%
	EGFR c.2369C>T; p.(T790M)	t_p	9.9	0.0%	5.9	0[d]	0.0%
UMCG-AB-007	*EGFR* c.2235_2249del; p.(E746_A750del)	t_0	5.1	0.0%	3.2	0[e]	0.0%
	EGFR c.2235_2249del; p.(E746_A750del)	t_1	7.8	0.0%	3.5	0[d]	0.0%
UMCG-AB-008	*EGFR* c.2235_2249del; p.(E746_A750del)	t_0	10.0	1.4%	5.9	361	23.0%
	EGFR c.2235_2249del; p.(E746_A750del)	t_1	8.6	0.0%	5.4	0[d]	0.0%
	KRAS c.38G>A; p.(G13D)	t_0	10.0	0.0%	5.9	0[b]	0.0%
	KRAS c.38G>A; p.(G13D)*	t_1	8.6	0.6%	10.8	0[b]	0.0%
UMCG-AB-009	*EGFR* c.2239_2256del; p.(L747_A750delinsP)	t_0	9.9	2.0%	4.2	3680	15.2%
	EGFR c.2239_2256del; p.(L747_A750delinsP)	t_1	10.0	0.1%	5.9	7	0.2%
	EGFR c.2239_2256del; p.(L747_A750delinsP)	t_p	10.0	1.7%	5.9	3550	16.2%
	EGFR c.2369C>T; p.(T790M)*	t_0	9.9	1.4%	8.4[†]	0[b]	0.0%
	EGFR c.2369C>T; p.(T790M)	t_1	10.0	0.0%	5.9	0[c]	0.0%
	EGFR c.2369C>T; p.(T790M)	t_p	10.0	0.9%	5.9	234	1.7%

UMCG-AB-010	*EGFR* c.2155G>T; p.(G719C)	t_0	2.9	0.0%	5.9	0[c]	0.0%
	EGFR c.2155G>T; p.(G719C)	t_1	4.5	0.0%	5.9	0[b]	0.0%
	EGFR c.2155G>T; p.(G719C)	t_p	3.7	0.0%	5.9	0[b]	0.0%
	KRAS c.34G>T; p. (G12C)	t_0	2.9	0.0%	5.9	0[c]	0.0%
	KRAS c.34G>T; p. (G12C)	t_1	4.5	0.0%	5.9	0[b]	0.0%
	KRAS c.34G>T; p. (G12C)	t_p	3.7	0.0%	5.9	0[d]	0.0%
	PIK3CA c.1624G>A; p.(E542K)	t_0	2.9	0.0%	5.9	0[c]	0.0%
	PIK3CA c.1624G>A; p.(E542K)*	t_1	4.5	0.0%	5.9	38	0.7%
	PIK3CA c.1624G>A; p.(E542K)	t_p	3.7	0.0%	5.9	0[e]	0.0%
UMCG-AB-011	*EGFR* c.2235_2249del; p.(E746_A750del)	t_0	N/A	2.3%	3.5	255	18.1%
	EGFR c.2235_2249del; p.(E746_A750del)	t_p	N/A	2.4%	5.9	801	24.0%
	EGFR c.2369C>T; p.(T790M)	t_0	N/A	2.1%	3.5	76	6.5%
	EGFR c.2369C>T; p.(T790M)	t_p	N/A	2.2%	5.9	291	8.9%
	EGFR c.2389T>A; p.(C797S)	t_0	N/A	0.0%	3.5	0[e]	0.0%
	EGFR c.2389T>A; p.(C797S)	t_p	N/A	0.5%	5.9	19	0.7%
UMCG-AB-012	*EGFR* c.2239_2256del; p.(L747_A750delinsP)	t_0	10.0	1.7%	5.9	427	10.1%
	EGFR c.2239_2256del; p.(L747_A750delinsP)	t_1	8.6	0.0%	5.9	0[d]	0.0%
	EGFR c.2239_2256del; p.(L747_A750delinsP)	t_p	4.6	0.6%	5.9	993	8.6%
	EGFR c.2369C>T; p.(T790M)	t_0	10.0	0.0%	5.9	0[c]	0.0%
	EGFR c.2369C>T; p.(T790M)	t_1	8.6	0.0%	5.9	0[d]	0.0%
	EGFR c.2369C>T; p.(T790M)	t_p	4.6	0.5%	5.9	6	2.4%
UMCG-AB-013	*EGFR* c.2156G>C; p.(G719A)	t_0	4.8	3.9%	3.4	458	10.0%
	EGFR c.2156G>C; p.(G719A)	t_1	6.1	2.2%	4.0	34	1.6%
UMCG-AB-014	*EGFR* c.2155G>T; p.(G719C)	t_0	2.5	0.7%	1.9	46	4.0%
	EGFR c.2155G>T; p.(G719C)	t_1	1.9	0.0%	3.6	0[e]	0.0%
	EGFR c.2303G>T; p.(S768I)	t_0	2.5	2.4%	1.9	45	3.2%
	EGFR c.2303G>T; p.(S768I)	t_1	1.9	0.0%	3.6	0[d]	0.0%
UMCG-AB-015	*EGFR* c.2235_2249del; p.(E746_A750del)	t_0	7.8	2.6%	3.9	804	30.7%
	EGFR c.2235_2249del; p.(E746_A750del)*	t_1	5.1	0.4%	6.2	0[c]	0.0%
	EGFR c.2235_2249del; p.(E746_A750del)	t_p	7.2	1.5%	4.5	58	3.0%
	EGFR c.2369C>T; p.(T790M)	t_0	7.8	2.7%	3.9	302	16.8%
	EGFR c.2369C>T; p.(T790M)	t_1	5.1	0.0%	7.1	0[c]	0.0%
	EGFR c.2369C>T; p.(T790M)	t_p	7.2	0.0%	4.5	0[e]	0.0%
UMCG-AB-018	*EGFR* c.2126A>C; p.(E709A)	t_0	10.0	3.1%	5.5	189	6.5%
	EGFR c.2126A>C; p.(E709A)	t_1	5.7	4.1%	4.1	230	6.6%
	EGFR c.2155G>A; p.(G719S)	t_0	10.0	1.1%	5.5	178	2.6%
	EGFR c.2155G>A; p.(G719S)	t_1	5.7	2.4%	4.1	167	5.7%
UMCG-AB-019	*EGFR* c.2236_2249del; p.(E746_A750del)	t_0	6.1	0.8%	5.9	103	3.8%
	EGFR c.2236_2249del; p.(E746_A750del)	t_1	6.0	1.3%	4.4	361	11.1%
UMCG-AB-020	*EGFR* c.2573T>G; p.(L858R)	t_0	10.0	4.0%	5.9	1893	27.5%
	EGFR c.2573T>G; p.(L858R)	t_1	10.0	4.4%	5.9	1316	13.8%
UMCG-AB-021	*BRAF* c.1799T>A; p.(V600E)	t_0	10.0	0.4%	11.8	4	0.2%
	BRAF c.1799T>A; p.(V600E)	t_1	10.0	1.2%	5.9	69	1.9%
UMCG-AB-023	*BRAF* c.1406G>C; p.(G469A)	t_0	9.9	5.0%	4.7	66	1.2%
	BRAF c.1406G>C; p.(G469A)	t_1	10.0	5.1%	5.9	57	0.9%

UMCG-AB-025	*BRAF* c.1406G>C; p.(G469A)	t_0	4.1	5.3%	4.3	443	24.8%
	BRAF c.1406G>C; p.(G469A)	t_1	4.4	5.2%	5.9	669	18.9%
	BRAF c.1406G>C; p.(G469A)	t_p	4.6	5.3%	5.9	1217	25.5%
UMCG-AB-026	*BRAF* c.1406G>T; p.(G469V)	t_0	3.5	1.1%	5.9	33	1.4%
	BRAF c.1406G>T; p.(G469V)	t_1	1.5	1.4%	5.9	21	2.0%
UMCG-AB-027	*BRAF* c.1799T>A; p.(V600E)	t_0	10.0	2.0%	5.9	217	7.3%
	BRAF c.1799T>A; p.(V600E)	t_1	10.0	1.7%	5.9	125	3.3%
	BRAF c.1799T>A; p.(V600E)	t_p	10.0	1.7%	5.9	163	3.1%
UMCG-AB-029	*BRAF* c.1799T>A; p.(V600E)*	t_0	10.0	0.4%	5.6[†]	0[d]	0.0%
	BRAF c.1799T>A; p.(V600E)	t_1	9.9	0.5%	5.9	19	0.4%
UMCG-AB-030	*BRAF* c.1799T>A; p.(V600E)	t_0	10.0	0.8%	5.0	17	0.7%
	BRAF c.1799T>A; p.(V600E)	t_1	10.1	0.0%	4.0	0[c]	0.0%
	BRAF c.1799T>A; p.(V600E)*	t_p	10.1	0.0%	5.3	24	0.9%
	PIK3CA c.1624G>A; p.(E542K)	t_0	10.0	0.0%	5.0	0[c]	0.0%
	PIK3CA c.1624G>A; p.(E542K)	t_1	10.1	0.0%	4.0	0[c]	0.0%
	PIK3CA c.1624G>A; p.(E542K)	t_p	10.1	0.0%	3.8	0[c]	0.0%
UMCG-AB-032	*PIK3CA* c.1624G>A; p.(E542K)	t_0	8.5	0.0%	5.9	0[c]	0.0%
	PIK3CA c.1624G>A; p.(E542K)	t_1	9.3	0.0%	5.9	0[c]	0.0%
	PIK3CA c.1624G>A; p.(E542K)	t_p	10.0	0.0%	5.9	0[b]	0.0%
UMCG-AB-036	*PIK3CA* c.1633G>A; p.(E545K)*	t_0	5.6	0.0%	3.7	9	0.6%
	PIK3CA c.1633G>A; p.(E545K)	t_1	6.6	0.0%	5.1	0[d]	0.0%
	PIK3CA c.1633G>A; p.(E545K)*	t_p	2.1	1.8%	4.4	0[c]	0.0%
UMCG-AB-038	*KRAS* c.38G>A; p.(G13D)	t_0	4.2	2.4%	4.4	3126	14.5%
	KRAS c.38G>A; p.(G13D)	t_1	1.8	3.9%	5.9	23877	20.4%
UMCG-AB-039	*KRAS* c.35G>C; p.(G12A)	t_0	4.4	0.0%	5.4	0[d]	0.0%
	KRAS c.35G>C; p.(G12A)	t_1	3.6	0.0%	5.9	0[b]	0.0%
	KRAS c.35G>C; p.(G12A)	t_p	5.3	0.0%	5.1	0[e]	0.0%
UMCG-AB-040	*KRAS* c.34G>C; p.(G12R)	t_0	N/A	0.0%	5.9	0[a]	0.0%
	KRAS c.34G>C; p.(G12R)	t_1	10	0.0%	5.6	0[b]	0.0%
UMCG-AB-042	*KRAS* c.35G>A; p.(G12D)	t_0	4.2	2.4%	5.9	845	4.9%
	KRAS c.35G>A; p.(G12D)	t_1	4.5	1.9%	5.9	378	3.8%
	PIK3CA c.1633G>A; p.(E545K)	t_0	4.2	1.8%	5.9	86	2.7%
	PIK3CA c.1633G>A; p.(E545K)	t_1	4.5	1.1%	5.9	16	5.4%
UMCG-AB-044	*KRAS* c.182A>T; p.(Q61L)	t_0	10.0	3.7%	5.9	837	17.9%
	KRAS c.182A>T; p.(Q61L)	t_1	10.0	3.7%	5.9	1120	12.1%
UMCG-AB-045	*KRAS* c.38G>A; p.(G13D)*	t_0	7.3	0.0%	5.9	37	1.5%
	KRAS c.38G>A; p.(G13D)	t_1	10.0	2.7%	5.9	172	4.0%
UMCG-AB-046	*KRAS* c.35G>A; p.(G12D)	t_0	10.0	4.6%	5.9	777	15.7%
	KRAS c.35G>A; p.(G12D)	t_1	10.0	0.0%	5.9	0[c]	0.0%
UMCG-AB-047	*KRAS* c.35G>T; p.(G12V)	t_0	3.6	0.0%	3.3	0[e]	0.0%
	KRAS c.35G>T; p.(G12V)	t_1	4.5	0.0%	4.5	0[d]	0.0%
	KRAS c.35G>T; p.(G12V)	t_p	4.9	0.0%	4.3	0[c]	0.0%
UMCG-AB-048	*KRAS* c.35G>T; p.(G12V)	t_0	2.9	1.6%	5.9	71	2.3%
	KRAS c.35G>T; p.(G12V)	t_1	2.6	0.0%	5.9	0[e]	0.0%
	KRAS c.35G>T; p.(G12V)	t_p	3.6	0.4%	5.9	164	1.2%

UMCG-AB-049	*KRAS* c.35G>A; p.(G12D)	t_0	7.8	1.3%	5.9	54	0.9%
	KRAS c.35G>A; p.(G12D)	t_1	10.0	1.5%	5.9	21	0.4%
	KRAS c.35G>A; p.(G12D)	t_p	10.0	4.4%	5.9	64	3.0%
	BRAF c.1406G>T; p.(G469V)	t_0	7.8	0.9%	11.8	14	0.3%
	BRAF c.1406G>T; p.(G469V)	t_1	10.0	1.5%	5.9	13	0.3%
	BRAF c.1406G>T; p.(G469V)	t_p	10.0	4.4%	5.9	144	3.4%
UMCG-AB-050	*KRAS* c.34G>T; p.(G12C)	t_0	10.0	0.3%	4.2	38	1.2%
	KRAS c.34G>T; p.(G12C)	t_1	10.0	0.0%	4.5	0[c]	0.0%
UMCG-AB-051	*KRAS* c.35G>T; p.(G12V)	t_0	10.0	0.0%	5.9	0[b]	0.0%
	KRAS c.35G>T; p.(G12V)	t_1	10.0	0.0%	5.6	0[b]	0.0%
	KRAS c.35G>T; p.(G12V)	t_p	7.8	0.0%	5.1	0[c]	0.0%
UMCG-AB-052	*KRAS* c.34G>T; p.(G12C)	t_0	10.0	1.3%	5.6	131	5.7%
	KRAS c.34G>T; p.(G12C)	t_1	10.0	1.4%	5.9	262	7.4%
	KRAS c.34G>T; p.(G12C)	t_p	9.9	1.4%	5.9	260	14.0%
UMCG-AB-053	*KRAS* c.35G>T; p.(G12V)	t_0	10.0	4.9%	5.9	443	11.6%
	KRAS c.35G>T; p.(G12V)	t_1	7.0	2.9%	5.9	228	7.5%
	KRAS c.35G>T; p.(G12V)	t_p	7.9	3.1%	4.0	218	10.8%
UMCG-AB-054	*KRAS* c.34G>T; p.(G12C)*	t_0	10.0	0.0%	2.8	18	1.2%
	KRAS c.34G>T; p.(G12C)	t_1	9.9	0.0%	2.1	0[d]	0.0%
UMCG-AB-055	*KRAS* c.35G>A; p.(G12D)	t_0	11.4	5.2%	5.9	3015	40.4%
	KRAS c.35G>A; p.(G12D)	t_1	6.8	0.6%	5.9	17	0.5%
UMCG-AB-056	*KRAS* c.35G>A; p.(G12D)	t_0	10.0	0.0%	5.6	0[c]	0.0%
	KRAS c.35G>A; p.(G12D)	t_1	10.1	0.0%	5.9	0[c]	0.0%
UMCG-AB-057	*KRAS* c.35G>A; p.(G12D)	t_0	4.6	3.5%	2.8	209	8.3%
	KRAS c.35G>A; p.(G12D)	t_1	4.3	2.4%	5.9	67	2.0%
	KRAS c.35G>A; p.(G12D)	t_p	3.7	2.4%	5.9	227	6.2%
UMCG-AB-059	*KRAS* c.34G>T; p.(G12C)	t_0	4.3	0.0%	5.9	0[c]	0.0%
	KRAS c.34G>T; p.(G12C)	t_1	4.6	0.6%	5.9	34	0.9%
	KRAS c.34G>T; p.(G12C)	t_p	4.7	0.6%	5.9	33	1.2%
UMCG-AB-060	*KRAS* c.34G>T; p.(G12C)	t_0	4.7	1.3%	4.1	21	2.4%
	KRAS c.34G>T; p.(G12C)	t_1	3.8	0.9%	2.7	31	3.4%
	KRAS c.34G>T; p.(G12C)	t_p	4.1	1.3%	3.6	33	2.9%

Grey shading was applied to distinguish between different variants in cases in which multiple variants were detected using UltraSEEK®. *Discordant cases. †Discordant results with lower input in ddPCR reaction compared to UltraSEEK. The overall concordance between UltraSEEK® and ddPCR was 92%, with PPA of 93% and NPA of 91%. For negative ddPCR results, the detection sensitivity for that specific assay is indicated as [a]<0.1-0.2%, [b]0.3-0.4%, [c]0.5-0.6%, [d]0.7-0.8%, or [e]0.9-1.0%. ddPCR, droplet digital polymerase chain reaction; VAF, variant allele frequency; t_0, plasma collected at the start of treatment; t_1, plasma collected at first response evaluation (4-6 weeks after treatment initiation); t_p, plasma collected at presentation of disease progression; N/A, not available.

Supplemental Table S4. Detection of tumor tissue-derived variants in the baseline plasma sample using the UltraSEEK® Lung Panel.

Patient ID	Tissue mutation[*] – VAF	Tumor cells (%)	UltraSEEK mutation – VAF	CcfDNA input (ng)	Conclusion
UMCG-AB-001	*EGFR* c.2240_2254del; p.(L747_T751del) – 8%	20%	*EGFR* c.2240_2254del – 0.5%	3.9	Concordant
UMCG-AB-002	*EGFR* c.2573T>G; p.(L858R) – 22%<br>*EGFR* c.2369C>T; p.(T790M) – 15%	20%	*EGFR* c.2573T>G – 5.0%<br>*EGFR* c.2369C>T – 2.9%	10.0	Concordant<br>Concordant
UMCG-AB-003	*EGFR* c.2573T>G; p.(L858R) – 42%<br>*EGFR* c.2369C>T; p.(T790M) – 22%	40%	*EGFR* c.2573T>G – 3.7%<br>*EGFR* c.2369C>T – 0.5%	5.7	Concordant<br>Concordant
UMCG-AB-004	*EGFR* c.2240_2254del; p.(L747_T751del) – 72%	40%	*EGFR* c.2240_2254del – 0.4%	9.9	Concordant
UMCG-AB-005	*EGFR* c.2237_2256delinsTC; p.(E746_S752delinsV)[†] – 43%	60%	Not detected	6.0	Not covered by UltraSEEK
UMCG-AB-006	*EGFR* c.2573T>G; p.(L858R) – 54%<br>*EGFR* c.2369C>T; p.(T790M) – 18%	50%	*EGFR* c.2573T>G – 2.9%<br>*EGFR* c.2369C>T – 0.4%	6.4	Concordant<br>Concordant
UMCG-AB-007	*EGFR* c.2235_2249del; p.(E746_A750del) – 43%	60%	Not detected	5.1	Missed by UltraSEEK
UMCG-AB-008	*EGFR* c.2235_2249del; p.(E746_A750del) – 66%	20%	*EGFR* c.2235_2249del – 1.4%	10.0	Concordant
UMCG-AB-009	*EGFR* c.2235_2256del; p.(L747_S752del)[‡] – 55%	70%	*EGFR* c.2239_2248delinsC – 2.0%	9.9	Calling of a different variant
UMCG-AB-010	*EGFR* c.2155G>T; p.(G719C) – 35%<br>*KRAS* c.34G>T; p. (G12C) – 32%<br>*PIK3CA* c.1624G>A; p.(E542K) – 24%	50%	Not detected<br>Not detected<br>Not detected	2.9	Missed by UltraSEEK<br>Missed by UltraSEEK<br>Missed by UltraSEEK
UMCG-AB-011	*EGFR* c.2235_2249del; p.(E746_A750del) – 90%<br>*EGFR* c.2369C>T; p.(T790M) – 38%	50%	*EGFR* c.2235_2249del – 2.3%<br>*EGFR* c.2369C>T – 2.1%	N/A	Concordant<br>Concordant
UMCG-AB-012	*EGFR* c.2239_2248delinsC; p.(L747_A750delinsP) – 8%	N/A	*EGFR* c.2239_2248delinsC – 1.7%	10.0	Concordant
UMCG-AB-013	*EGFR* c.2156G>C; p.(G719A) – 80%<br>Not detected	60%	*EGFR* c.2156G>C – 3.9%<br>*PIK3CA* c.3140A>G – 1.6%	4.8	Concordant<br>Only detected by UltraSEEK
UMCG-AB-014	*EGFR* c.2155G>T; p.(G719C) – 26%<br>*EGFR* c.2303G>T; p.(S768I) – 26%	80%	*EGFR* c.2155G>T – 0.7%<br>*EGFR* c.2303G>T – 2.4%	2.5	Concordant<br>Concordant
UMCG-AB-015	*EGFR* c.2235_2249del; p.(E746_A750del) – 20%<br>*EGFR* c.2369C>T; p.(T790M) – 11%	30%	*EGFR* c.2235_2249del – 2.6%<br>*EGFR* c.2369C>T – 2.7%	7.8	Concordant<br>Concordant
UMCG-AB-016	*EGFR* c.2310_2311insGGG; p.(D770_N771insG)[†] – 20%	30%	Not detected	7.4	Not covered by UltraSEEK
UMCG-AB-017	*EGFR* c.2316_2321dup; p.(H773_V774dup)[†] – 15%	30%	Not detected	10.0	Not covered by UltraSEEK
UMCG-AB-018	*EGFR* c.2126A>C; p.(E709A) – 24%<br>*EGFR* c.2155G>A; p.(G719S) – 12%	20%	*EGFR* c.2126A>C – 3.1%<br>*EGFR* c.2155G>A – 1.1%	10.0	Concordant<br>Concordant
UMCG-AB-020	*EGFR* c.2573T>G; p.(L858R) – 83%	25%	*EGFR* c.2573T>G – 4.0%	10.0	Concordant
UMCG-AB-021	*BRAF* c.1799T>A; p.(V600E) – 14%	30%	*BRAF* c.1799T>A – 0.4%	10.0	Concordant
UMCG-AB-022	*BRAF* c.1782A>G; p.(D594G) – 11%	40%	Not detected	9.9	Missed by UltraSEEK
UMCG-AB-023	*BRAF* c.1406G>C; p.(G469A) – 11%	20%	*BRAF* c.1406G>C – 5.0%	9.9	Concordant
UMCG-AB-025	*BRAF* c.1406G>C; p.(G469A) – 51%	30%	*BRAF* c.1406G>C – 5.3%	4.1	Concordant
UMCG-AB-026	*BRAF* c.1406G>T; p.(G469V) – 39%	50%	*BRAF* c.1406G>T – 1.1%	3.5	Concordant
UMCG-AB-027	*BRAF* c.1799T>A; p.(V600E) – 27%	80%	*BRAF* c.1799T>A – 2.0%	10.0	Concordant
UMCG-AB-028	*BRAF* c.1397G>T; p.(G466V)[†] – 11%	20%	Not detected	10.0	Not covered by UltraSEEK
UMCG-AB-029	*BRAF* c.1799T>A; p.(V600E) – 26%	60%	*BRAF* c.1799T>A – 0.4%	10.0	Concordant
UMCG-AB-030	*BRAF* c.1799T>A; p.(V600E) – 18%<br>*PIK3CA* c.1624G>A; p.(E542K) – 20%	50%	*BRAF* c.1799T>A – 0.8%<br>Not detected	10.0	Concordant<br>Missed by UltraSEEK
UMCG-AB-031	*BRAF* c.1799_1801del; p.(V600_K601delinsE)[†] – 29%	30%	Not detected	10.0	Not covered by UltraSEEK
UMCG-AB-032	*PIK3CA* c.1624G>A; p.(E542K) – 24%	30%	Not detected	8.5	Missed by UltraSEEK
UMCG-AB-034	None detected	50%	None detected	3.0	Concordant
UMCG-AB-037	None detected	20%	None detected	10.0	Concordant
UMCG-AB-038	*KRAS* c.38G>A; p.(G13D) – 35%	20%	*KRAS* c.38G>A – 2.5%	4.2	Concordant

UMCG-AB-039	*KRAS* c.35G>C; p.(G12A) – 27%	30%	Not detected	4.4	Missed by UltraSEEK
UMCG-AB-040	*KRAS* c.34G>C; p.(G12R) – 9%	60%	Not detected	N/A	Missed by UltraSEEK
UMCG-AB-041	*KRAS* c.34_35delinsTT; p.(G12F)‡ – 77%	40%	*KRAS* c.34G>T – 0.7%	10.0	Calling of a different variant
UMCG-AB-042	*KRAS* c.35G>A; p.(G12D) – 15%<br>*PIK3CA* c.1633G>A; p.(E545K) – 10%	20%	*KRAS* c.35G>A – 2.4%<br>*PIK3CA* c.1633G>A – 1.8%	4.2	Concordant<br>Concordant
UMCG-AB-043	*KRAS* c.34_35delinsTT; p.(G12F)† – 62%	60%	Not detected	9.9	Not covered by UltraSEEK
UMCG-AB-044	*KRAS* c.182A>T; p.(Q61L) – 5%	20%	*KRAS* c.182A>T – 3.7%	10.0	Concordant
UMCG-AB-045	*KRAS* c.38G>A; p.(G13D) – 40%	60%	Not detected	7.3	Missed by UltraSEEK
UMCG-AB-046	*KRAS* c.35G>A; p.(G12D) – 27%	70%	*KRAS* c.35G>A – 4.6%	10.0	Concordant
UMCG-AB-047	*KRAS* c.35G>T; p.(G12V) – 30%	30%	Not detected	3.6	Missed by UltraSEEK
UMCG-AB-048	*KRAS* c.35G>T; p.(G12V) – 23%	30%	*KRAS* c.35G>T – 1.6%	2.9	Concordant
UMCG-AB-049	*KRAS* c.35G>A; p.(G12D) – 30%<br>*BRAF* c.1406G>T; p.(G469V) – 28%	30%	*KRAS* c.35G>A – 1.3%<br>*BRAF* c.1406G>T – 0.9%	7.8	Concordant<br>Concordant
UMCG-AB-050	*KRAS* c.34G>T; p.(G12C) – 69%	50%	*KRAS* c.34G>T – 0.3%	10.0	Concordant
UMCG-AB-051	*KRAS* c.35G>T; p.(G12V) – N/A	20%	Not detected	10.0	Missed by UltraSEEK
UMCG-AB-052	*KRAS* c.34G>T; p.(G12C) – 42%	80%	*KRAS* c.34G>T – 1.3%	10.0	Concordant
UMCG-AB-053	*KRAS* c.35G>T; p.(G12V) – 51%	40%	*KRAS* c.35G>T – 4.9%	10.0	Concordant
UMCG-AB-054	*KRAS* c.34G>T; p.(G12C) – 25%	50%	Not detected	10.0	Missed by UltraSEEK
UMCG-AB-055	*KRAS* c.35G>A; p.(G12D) – 53%	30%	*KRAS* c.35G>A – 5.2%	11.4	Concordant
UMCG-AB-056	*KRAS* c.35G>A; p.(G12D) – 27%	40%	Not detected	10.0	Missed by UltraSEEK
UMCG-AB-057	*KRAS* c.35G>A; p.(G12D) – N/A	30%	*KRAS* c.35G>A – 3.5%	4.6	Concordant
UMCG-AB-058	*KRAS* c.33_35delinsCGT; p.(G12V)† – 16%	70%	Not detected	10.0	Not covered by UltraSEEK
UMCG-AB-059	*KRAS* c.34G>T; p.(G12C) – 20%	30%	Not detected	4.3	Missed by UltraSEEK
UMCG-AB-060	*KRAS* c.34G>T; p.(G12C) – 26%	30%	*KRAS* c.34G>T – 1.3%	4.7	Concordant
UKE-AB-001	*EGFR* c.2573T>G; p.(L858R) – N/A<br>*PIK3CA* c.1633G>A; p.(E545K) – N/A<br>*BRAF* c.1406G>C; p.(G469A) – N/A	>20%	*EGFR* c.2573T>G – 0.5%<br>*PIK3CA* c.1633G>A – 0.8%<br>Not detected	N/A	Concordant<br>Concordant<br>Missed by UltraSEEK
UKE-AB-002	*KRAS* c.34G>T; p.(G12C) – N/A	>20%	*KRAS* c.34G>T – 0.9%	4.4	Concordant
UKE-AB-003	*EGFR* c.2311_2319dup; p.(H773_V774insNPH) – N/A	>20%	Not detected	N/A	Missed by UltraSEEK
UKE-AB-004	*PIK3CA* c.1624G>C; p.(E542Q)† – N/A<br>Not detected	>20%	Not detected<br>*EGFR* c.2239_2256del – 0.8%	N/A	Not covered by UltraSEEK<br>Only detected by UltraSEEK
UKE-AB-005	*PIK3CA* c.1633G>A; p.(E545K) – N/A	>20%	Not detected	9.7	Missed by UltraSEEK
UKE-AB-006	*EGFR* c.2235_2249del; p.(E746_A750del) – N/A<br>*EGFR* c.2369C>T; p.(T790M) – N/A	>20%	*EGFR* c.2235_2249del – 1.6%<br>*EGFR* c.2369C>T – 1.4%	4.4	Concordant<br>Concordant
UKE-AB-007	None detected	>20%	None detected	2.9	Concordant
UKE-AB-008	*KRAS* c.33_34delinsCT; p.(G12C)†– N/A	>20%	Not detected	4.7	Not covered by UltraSEEK
UKE-AB-009	*BRAF* c.1799T>A; p.(V600E) – N/A	>20%	*BRAF* c.1799T>A – 1.8%	8.9	Concordant
UKE-AB-011	*KRAS* c.182A>T; p.(Q61L) – N/A<br>Not detected	>20%	*KRAS* c.182A>T – 0.8%<br>*EGFR* c.2310_2311insGGT – 1.3%	8.9	Concordant<br>Only detected by UltraSEEK
UKE-AB-012	None detected	>20%	None detected	10.0	Concordant

Tumor variant allele frequency and tumor cell percentage (which is at least 20% according to local standard operating procedures) were not available for UKE samples as these were not reported during routine diagnostics. *Mutations as reported in the pathology registries. Clinically relevant tumor-specific mutations were determined by routine diagnostic NGS performed in the ISO15189-accredited laboratory with various hotspot panels following Dutch guidelines (https://richtlijnendatabase.nl/richtlijn/niet_kleincellig_longcarcinoom/startpagina_-_niet-kleincellig_longcarcinoom.html, accessed on 14 June 2023) for predictive testing of tissue biopsies from patients with metastasized NSCLC including *EGFR*, *BRAF*, *KRAS*, *PIK3CA*, *ALK* and *ROS1* and reported in the Dutch Pathology Registry (https://www.palga.nl/en/public-database.html, accessed on 14 June 2023). †Mutation not covered by the UltraSEEK® Lung Panel. ‡A gene variant was detected in the genomic region, however had a different annotation of the same gene called by the UltraSEEK® Lung Panel. VAF, variant allele frequency; N/A, not available.

Supplemental Table S5. Validation of undetected tumor-derived variants in baseline plasma using UltraSEEK® with mutation-specific ddPCR.

Patient ID	Tissue mutation – VAF	Timepoint	UltraSEEK mutation – VAF	ddPCR mutation – VAF	Conclusion
UMCG-AB-007	*EGFR* c.2235_2249del; p.(E746_A750del) – 43%	t_0	Not detected	Not detected	Concordant
UMCG-AB-010	*EGFR* c.2155G>T; p.(G719C) – 35%<br>*KRAS* c.34G>T; p. (G12C) – 32%<br>*PIK3CA* c.1624G>A; p.(E542K) – 24%	t_0<br>t_0<br>t_0	Not detected<br>Not detected<br>Not detected	Not detected<br>Not detected<br>Not detected	Concordant<br>Concordant<br>Concordant
UMCG-AB-030	*BRAF* c.1799T>A; p.(V600E) – 18%<br>*PIK3CA* c.1624G>A; p.(E542K) – 20%	t_0<br>t_0	*BRAF* c.1799T>A – 0.8%<br>Not detected	*BRAF* c.1799T>A – 0.7%<br>Not detected	Concordant<br>Concordant
UMCG-AB-032	*PIK3CA* c.1624G>A; p.(E542K) – 24%	t_0	Not detected	Not detected	Concordant
UMCG-AB-039	*KRAS* c.35G>C; p.(G12A) – 27%	t_0	Not detected	Not detected	Concordant
UMCG-AB-040	*KRAS* c.34G>C; p.(G12R) – 9%	t_0	Not detected	Not detected	Concordant
UMCG-AB-045	*KRAS* c.38G>A; p.(G13D) – 40%	t_0	Not detected	*KRAS* c.38G>A – 1.5%	Missed by UltraSEEK
UMCG-AB-047	*KRAS* c.35G>T; p.(G12V) – 30%	t_0	Not detected	Not detected	Concordant
UMCG-AB-051	*KRAS* c.35G>T; p.(G12V) – N/A	t_0	Not detected	Not detected	Concordant
UMCG-AB-054	*KRAS* c.34G>T; p.(G12C) – 25%	t_0	Not detected	*KRAS* c.34G>T – 1.2%	Missed by UltraSEEK
UMCG-AB-056	*KRAS* c.35G>A; p.(G12D) – 27%	t_0	Not detected	Not detected	Concordant
UMCG-AB-059	*KRAS* c.34G>T; p.(G12C) – 20%	t_0	Not detected	Not detected	Concordant

Only mutations covered by the UltraSEEK® Lung Panel were included in this table. Five samples (UMCG-AB-022, UKE-AB-001, UKE-AB-003, UKE-AB-004, UKE-AB-005) were excluded as no ddPCR analysis was performed. VAF, variant allele frequency; t_0, plasma collected at the start of treatment; N/A, not available.

Supplemental Table S6. Stepwise Cox regression analysis depicting the discriminators for PFS and OS.

Proxy added to model	Cut-off	-2Log Likelihood for PFS	Hazard ratio	*P*-value	-2Log Likelihood for OS	Hazard ratio	*P*-value
UltraSEEK VAF (%)	≥10% decrease	208.7	0.48	0.040	194.1	0.42	0.023
UltraSEEK VAF (%)	≥15% decrease	208.7	0.48	0.040	194.1	0.42	0.023
UltraSEEK VAF (%)	≥31% decrease	210.3	0.55	0.103	196.0	0.49	0.066
ddPCR mutant molecules	≥31% decrease	203.3	0.32	0.002	192.5	0.38	0.009
ddPCR VAF (%)	≥31% decrease	208.3	0.46	0.031	195.6	0.49	0.049

P-values as <0.05 are considered significant. ddPCR, droplet digital polymerase chain reaction.

Supplemental Table S7. Comparison of ctDNA dynamics between UltraSEEK® and ddPCR.

Patient ID	Mutation of interest*	Durable response	Best response	ctDNA dynamics UltraSEEK	ctDNA dynamics ddPCR
UMCG-AB-001†	*EGFR* c.2240_2254del; p.(L747_T751del)	Durable responder (PFS = 48 weeks)	SD	t_0: 0.5% – t_1: 0.0% (-100%)	t_0: 0 – t_1: 0 (not detected)
UMCG-AB-003	*EGFR* c.2573T>G; p.(L858R)<br>*EGFR* c.2369C>T; p.(T790M)	Non-responder (PFS = 6 weeks)	PD	t_0: 3.7% – t_1: 3.7% (±0%)<br>t_0: 0.5% – t_1: 2.2% (+340%)	t_0: 207 – t_1: 546 (+163%)<br>t_0: 0 – t_1: 109 (+100%)
UMCG-AB-007	*EGFR* c.2235_2249del; p.(E746_A750del)	Durable responder (PFS = 135 weeks)	PR	t_0: 0.0% – t_1: 0.0% (not detected)	t_0: 0 – t_1: 0 (not detected)
UMCG-AB-008‡	*EGFR* c.2235_2249del; p.(E746_A750del)<br>*KRAS* c.38G>A; p.(G13D)	Durable responder (PFS = 35 weeks)	PR	t_0: 1.4% – t_1: 0.0% (-100%)<br>t_0: 0.0% – t_1: 0.6% (+100%)	t_0: 361 – t_1: 0 (-100%)<br>t_0: 0 – t_1: 0 (not detected)
UMCG-AB-009	*EGFR* c.2235_2249del; p.(E746_A750del)<br>*EGFR* c.2369C>T; p.(T790M)	Non-responder (PFS = 11 weeks)	PD	t_0: 2.0% – t_1: 0.1% (-95%)<br>t_0: 1.4% – t_1: 0.0% (-100%)	t_0: 3680 – t_1: 7 (-100%)<br>t_0: 0 – t_1: 0 (not detected)
UMCG-AB-010	*PIK3CA* c.1624G>A; p.(E542K)<br>*EGFR* c.2155G>T; p.(G719C)<br>*KRAS* c.34G>T; p.(G12C)	Durable responder (PFS = 42 weeks)	PR	t_0: 0.0% – t_1: 0.0% (not detected)<br>t_0: 0.0% – t_1: 0.0% (not detected)<br>t_0: 0.0% – t_1: 0.0% (not detected)	t_0: 0 – t_1: 38 (+100)<br>t_0: 0 – t_1: 0 (not detected)<br>t_0: 0 – t_1: 0 (not detected)
UMCG-AB-012	*EGFR* c.2239_2248delinsC; p.(L747_A750delinsP)	Durable responder (PFS = 43 weeks)	CR	t_0: 1.7% – t_1: 0.0% (-100%)	t_0: 427 – t_1: 0 (-100%)
UMCG-AB-013	*EGFR* c.2156G>C; p.(G719A)	Durable responder (PFS = 46 weeks)	PR	t_0: 3.9% – t_1: 2.2% (-44%)	t_0: 158 – t_1: 34 (-78%)
UMCG-AB-014	*EGFR* c.2155G>T; p.(G719C)<br>*EGFR* c.2303G>T; p.(S768I)	Durable responder (PFS = 145 weeks)	PR	t_0: 0.7% – t_1: 0.0% (-100%)<br>t_0: 2.4% – t_1: 0.0% (-100%)	t_0: 46 – t_1: 0 (-100%)<br>t_0: 45 – t_1: 0 (-100%)
UMCG-AB-015	*EGFR* c.2235_2249del; p.(E746_A750del)<br>*EGFR* c.2369C>T; p.(T790M)	Non-responder (PFS = 21 weeks)	PD	t_0: 2.6% – t_1: 0.4% (-85%)<br>t_0: 2.7% – t_1: 0.0% (-100%)	t_0: 804 – t_1: 0 (-100%)<br>t_0: 302 – t_1: 0 (-100%)
UMCG-AB-018	*EGFR* c.2126A>C; p.(E709A)<br>*EGFR* c.2155G>A; p.(G719S)	Non-responder (PFS = 6 weeks)	PD	t_0: 3.1% – t_1: 4.1% (+32%)<br>t_0: 1.1% – t_1: 2.4% (+118%)	t_0: 189 – t_1: 230 (+22%)<br>t_0: 178 – t_1: 167 (-6%)
UMCG-AB-019	*EGFR* c.2235_2249del; p.(E746_A750del)	Non-responder (PFS = 3 weeks)	PD	t_0: 0.8% – t_1: 1.3% (+63%)	t_0: 103 – t_1: 361 (+250%)
UMCG-AB-020	*EGFR* c.2573T>G; p.(L858R)	Non-responder (PFS = 4 weeks)	PD	t_0: 4.0% – t_1: 4.4% (+10%)	t_0: 1893 – t_1: 1316 (-30%)
UMCG-AB-021	*BRAF* c.1799T>A; p.(V600E)	Non-responder (PFS = 6 weeks)	PD	t_0: 0.4% – t_1: 1.2% (+200%)	t_0: 4 – t_1: 69 (+1724%)
UMCG-AB-023	*BRAF* c.1406G>C; p.(G469A)	Non-responder (PFS = 4 weeks)	PD	t_0: 5.0% – t_1: 5.1% (+2%)	t_0: 66 – t_1: 57 (-14%)
UMCG-AB-025	*BRAF* c.1406G>C; p.(G469A)	Non-responder (PFS = 12 weeks)	SD	t_0: 5.3% – t_1: 5.2% (-2%)	t_0: 443 – t_1: 669 (+51%)
UMCG-AB-026†	*BRAF* c.1406G>T; p.(G469V)	Durable responder (PFS = 119 weeks)	PR	t_0: 1.1% – t_1: 1.4% (+27%)	t_0: 33 – t_1: 21 (-38%)
UMCG-AB-027	*BRAF* c.1799T>A; p.(V600E)	Non-responder (PFS = 20 weeks)	SD	t_0: 2.0% – t_1: 1.7% (-15%)	t_0: 217 – t_1: 125 (-42%)
UMCG-AB-029	*BRAF* c.1799T>A; p.(V600E)	Non-responder (PFS = 11 weeks)	SD	t_0: 0.4% – t_1: 0.5% (+25%)	t_0: 0 – t_1: 19 (+100%)

UMCG-AB-030	*BRAF* c.1799T>A; p.(V600E)<br>*PIK3CA* c.1624G>A; p.(E542K)	Durable responder (PFS = 97 weeks)	PR	t_0: 0.8% – t_1: 0.0% (-100%)<br>t_0: 0.0% – t_1: 0.0% (not detected)	t_0: 17 – t_1: 0 (-100%)<br>t_0: 0 – t_1: 0 (not detected)
UMCG-AB-032	*PIK3CA* c.1624G>A; p.(E542K)	Durable responder (PFS = 28 weeks)	SD	t_0: 0.0% – t_1: 0.0% (not detected)	t_0: 0 – t_1: 0 (not detected)
UMCG-AB-036†	*PIK3CA* c.1633G>A; p.(E545K)	Durable responder (PFS = 112 weeks)	SD	t_0: 0.0% – t_1: 0.0% (not detected)	t_0: 9 – t_1: 0 (-100%)
UMCG-AB-038	*KRAS* c.38G>A; p.(G13D)	Non-responder (PFS = 1 weeks)	PD	t_0: 2.4% – t_1: 3.9% (+63%)	t_0: 3126 – t_1: 23877 (+664%)
UMCG-AB-040	*KRAS* c.34G>C; p.(G12R)	Non-responder (PFS = 6 weeks)	PD	t_0: 0.0% – t_1: 0.0% (not detected)	t_0: 0 – t_1: 0 (not detected)
UMCG-AB-041	*KRAS* c.34G>T; p.(G12C)	Durable responder (PFS = 155 weeks)	PR	t_0: 0.7% – t_1: 0.2% (-71%)	N/A
UMCG-AB-042	*KRAS* c.35G>A; p.(G12D)<br>*PIK3CA* c.1633G>A; p.(E545K)	Durable responder (PFS = 219 weeks)	PR	t_0: 2.4% – t_1: 1.9% (-21%)<br>t_0: 1.8% – t_1: 1.1% (-39%)	t_0: 845 – t_1: 378 (-55%)<br>t_0: 86 – t_1: 16 (-81%)
UMCG-AB-044	*KRAS* c.182A>T; p.(Q61L)	Durable responder (PFS = 27 weeks)	PR	t_0: 3.7% – t_1: 3.7% (±0%)	t_0: 837 – t_1: 1120 (+34%)
UMCG-AB-045	*KRAS* c.38G>A; p.(G13D)	Durable responder (PFS = 127 weeks)	PR	t_0: 0.0% – t_1: 2.7% (+100%)	t_0: 37 – t_1: 172 (+367%)
UMCG-AB-046	*KRAS* c.35G>A; p.(G12D)	Durable responder (PFS = 217 weeks)	PR	t_0: 4.6% – t_1: 0.0% (-100%)	t_0: 777 – t_1: 0 (-100%)
UMCG-AB-047	*KRAS* c.35G>T; p.(G12V)	Non-responder (PFS = 6 weeks)	PD	t_0: 0.0% – t_1: 0.0% (not detected)	t_0: 0 – t_1: 0 (not detected)
UMCG-AB-048	*KRAS* c.35G>T; p.(G12V)	Durable responder (PFS = 50 weeks)	SD	t_0: 1.6% – t_1: 0.0% (-100%)	t_0: 71 – t_1: 0 (-100%)
UMCG-AB-049†	*KRAS* c.35G>A; p.(G12D)<br>*BRAF* c.1406G>T; p.(G469V)	Durable responder (PFS = 86 weeks)	PR	t_0: 1.3% – t_1: 1.5% (+15%)<br>t_0: 0.9% – t_1: 1.5% (+67%)	t_0: 54 – t_1: 21 (-62%)<br>t_0: 14 – t_1: 13 (-9%)
UMCG-AB-050	*KRAS* c.34G>T; p.(G12C)	Durable responder (PFS = 198 weeks)	CR	t_0: 0.3% – t_1: 0.0% (-100%)	t_0: 38 – t_1: 0 (-100%)
UMCG-AB-051	*KRAS* c.35G>T; p.(G12V)	Non-responder (PFS = 13 weeks)	PD	t_0: 0.0% – t_1: 0.0% (not detected)	t_0: 0 – t_1: 0 (not detected)
UMCG-AB-052	*KRAS* c.34G>T; p.(G12C)	Non-responder (PFS = 6 weeks)	PD	t_0: 1.3% – t_1: 1.4% (+8%)	t_0: 131 – t_1: 262 (+100%)
UMCG-AB-053	*KRAS* c.35G>T; p.(G12V)	Non-responder (PFS = 6 weeks)	PD	t_0: 4.9% – t_1: 2.9% (-41%)	t_0: 443 – t_1: 228 (-48%)
UMCG-AB-054†	*KRAS* c.34G>T; p.(G12C)	Durable responder (PFS = 154 weeks)	CR	t_0: 0.0% – t_1: 0.0% (not detected)	t_0: 18 – t_1: 0 (-100%)
UMCG-AB-055	*KRAS* c.35G>A; p.(G12D)	Durable responder (PFS = 149 weeks)	PR	t_0: 5.2% – t_1: 0.6% (-88%)	t_0: 3015 – t_1: 17 (-99%)
UMCG-AB-056	*KRAS* c.35G>A; p.(G12D)	Non-responder (PFS = 15 weeks)	SD	t_0: 0.0% – t_1: 0.0% (not detected)	t_0: 0 – t_1: 0 (not detected)
UMCG-AB-057	*KRAS* c.35G>A; p.(G12D)	Durable responder (PFS = 43 weeks)	PR	t_0: 3.5% – t_1: 2.4% (-31%)	t_0: 209 – t_1: 67 (-68%)
UMCG-AB-059	*KRAS* c.34G>T; p.(G12C)	Non-responder (PFS = 6 weeks)	PD	t_0: 0.0% – t_1: 0.6% (+100%)	t_0: 0 – t_1: 34 (+100%)
UMCG-AB-060†	*KRAS* c.34G>T; p.(G12C)	Non-responder (PFS = 6 weeks)	PD	t_0: 1.3% – t_1: 0.9% (-31%)	t_0: 21 – t_1: 31 (+47%)

UKE-AB-001	*EGFR* c.2573T>G; p.(L858R)<br>*PIK3CA* c.1633G>A; p.(E545K)<br>*BRAF* c.1406G>C; p.(G469A)	Durable responder (PFS = 39 weeks)	SD	t_0: 0.5% – t_1: 1.0% (+100%)<br>t_0: 0.8% – t_1: 1.7% (+113%)<br>t_0: 0.0% – t_1: 0.0% (not detected)	N/A
UKE-AB-002	*KRAS* c.34G>T; p.(G12C)	Non-responder (PFS = 15 weeks)	PD	t_0: 0.9% – t_1: 0.3% (-67%)	N/A
UKE-AB-004‡	*EGFR* c.2239_2248delinsC; p.(L747_A750delinsP)<br>*EGFR* c.2310_2311insGGT; p.(D770_N771insG)	Non-responder (PFS = 21 weeks)	PR	t_0: 0.8% – t_1: 0.0% (-100%)<br>t_0: 0.0% – t_1: 0.7% (+100%)	N/A
UKE-AB-008	*KRAS* c.34G>T; p.(G12C)	Durable responder (PFS = 189 weeks)	SD	t_0: 0.0% – t_1: 0.5% (+100%)	N/A
UKE-AB-011‡	*KRAS* c.182A>T; p.(Q61L)<br>*EGFR* c.2310_2311insGGT; p.(D770_N771insG)<br>*ERBB2* c.2326_2327insTGT; p.(G776delinsVC)	Durable responder (PFS = 140 weeks)	PR	t_0: 0.8% – t_1: 0.0% (-100%)<br>t_0: 1.3% – t_1: 0.0% (-100%)<br>t_0: 0.0% – t_1: 2.0% (+100%)	N/A

All patients for whom UltraSEEK® and ddPCR analysis were performed are displayed (*n*=41). In addition, seven cases positive with UltraSEEK® for which no ddPCR assay was available are included as well. The remaining ctDNA negative patients are excluded from this table (*n*=17; see Supplemental Table 4). A decrease in variant allele frequency (VAF) of ≥15% and mutant copy levels of ≥31% were considered a true decrease (blue); any other observation was compiled in the no decrease group (red). In cases with contradictory ctDNA dynamics, only the highest VAF or mutant copy levels were considered, and other variants were neglected (grey). Durable response is defined as a PFS >26 weeks. *Based on UltraSEEK® analysis or tumor tissue NGS (when covered by UltraSEEK®). †Discordant cases. ‡Cases with mixed ctDNA dynamics as determined by UltraSEEK®. PFS, progression-free survival; CR, complete response; PR, partial response; SD, stable disease; PD, progressive disease; t_0, plasma collected at the start of treatment; t_1, plasma collected at first response evaluation (4-6 weeks after treatment initiation); t_p, plasma collected at presentation of disease progression; N/A, not available.

Supplemental Table S8. Detection of acquired resistance mutations using the UltraSEEK® Lung Panel.

**Patient ID**	**Timepoint**	**Plasma mutation 1 – VAF**	**Plasma mutation 2 – VAF**	**Plasma mutation 3 – VAF**
UMCG-AB-002	t_0<br>t_p	*EGFR* c.2573T>G; p.(L858R) – 5.0%<br>*EGFR* c.2573T>G; p.(L858R) – 5.5%	*EGFR* c.2369C>T; p.(T790M) – 2.9%<br>*EGFR* c.2369C>T; p.(T790M) – 2.2%	*EGFR* c.2389T>A; p.(C797S) – ND<br>*EGFR* c.2389T>A; p.(C797S) – 4.2%
UMCG-AB-004	t_0<br>t_p	*EGFR* c.2240_2254del; p.(L747_T751del) – 0.4%<br>*EGFR* c.2240_2254del; p.(L747_T751del) – 2.0%	*EGFR* c.2369C>T; p.(T790M) – ND<br>*EGFR* c.2369C>T; p.(T790M) – 2.1%	
UMCG-AB-011	t_0<br>t_p	*EGFR* c.2235_2249del; p.(E746_A750del) – 2.3%<br>*EGFR* c.2235_2249del; p.(E746_A750del) – 2.4%	*EGFR* c.2369C>T; p.(T790M) – 2.1%<br>*EGFR* c.2369C>T; p.(T790M) – 2.2%	*EGFR* c.2389T>A; p.(C797S) – ND<br>*EGFR* c.2389T>A; p.(C797S) – 0.5%
UMCG-AB-012	t_0<br>t_1<br>t_p	*EGFR* c.2235_2248delinsC; p.(L747_A750delinsP) – 1.7%<br>*EGFR* c.2235_2248delinsC; p.(L747_A750delinsP) – ND<br>*EGFR* c.2235_2248delinsC; p.(L747_A750delinsP) – 0.6%	*EGFR* c.2369C>T; p.(T790M) – ND<br>*EGFR* c.2369C>T; p.(T790M) – ND<br>*EGFR* c.2369C>T; p.(T790M) – 0.5%	

VAF, variant allele frequency; t_0, plasma collected at the start of treatment; t_1, plasma collected at first response evaluation (4-6 weeks after treatment initiation); t_p, plasma collected at presentation of disease progression; ND, not detected.

Supplemental Table S9. Primer and probe sequences of the custom ddPCR assays.

Assay	Forward primer	Reverse primer	Mutant probe	Wildtype probe	Tm
EGFR exon19 deletion*	5'-GTGAGAAAGTTAAAATTCCCGTC-3'	5'-CACACAGCAAAGCAGAAAC-3'	5'-AAGGAATTAAGAGAAGCAACATCTCC-3'	5'-ATCGAGGATTTCCTTGTTGGCT-3'	60°C
EGFR C797S (c.2389T>A)	5'-GCCTGCTGGGCATCTG-3'	5'-TCTTTGTGTTCCCGGACATAGTC-3'	5'-TTCGGCAGCCTCCTG-3'	5'-TTCGGCTGCCTCCTG-3'	55°C
EGFR C797S (c.2390G>C)	5'-GCCTGCTGGGCATCTG-3'	5'-TCTTTGTGTTCCCGGACATAGTC-3'	5'-TTCGGCTCCCTCCTG-3'	5'-TTCGGCTGCCTCCTG-3'	55°C
EGFR L858R	5'-GCAGCATGTCAAGATCACAGATT-3'	5'-CCTCCTTCTGCATGGTATTCTTTCT-3'	5'-AGTTTGGCCCGCCCAA-3'	5'-AGTTTGGCCAGCCCAA-3'	60°C
EGFR T790M	5'-GCCTGCTGGGCATCTG-3'	5'-TCTTTGTGTTCCCGGACATAGTC-3'	5'-ATGAGCTGCATGATGAG-3'	5'-ATGAGCTGCGTGATGAG-3'	55°C

*Screening assay detecting multiple EGFR exon19 deletions. A primer input of 300nM in combination with 200nM probes was applied for all assays. For wet-lab validated commercially available ddPCR assays see Supplemental Table 1. Tm, melting temperature.

Supplemental Figures

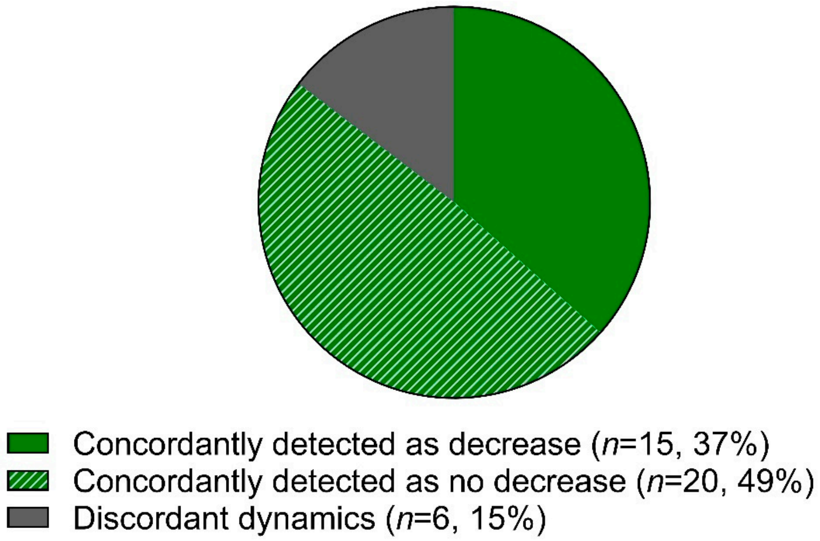

Supplemental Figure 1. Concordance of ctDNA dynamics between UltraSEEK® and ddPCR.

Supplemental References

1. Leest, P. van der; Boonstra, P.A.; Elst, A. ter; Kempen, L.C. van; Tibbesma, M.; Koopmans, J.; Miedema, A.; Tamminga, M.; Groen, H.J.M.; Reyners, A.K.L.; et al. Comparison of Circulating Cell-Free DNA Extraction Methods for Downstream Analysis in Cancer Patients. *Cancers (Basel)* **2020**, *12*, 1222.
2. Boonstra, P.A.; Elst, A. ter; Tibbesma, M.; Bosman, L.J.; Mathijssen, R.; Atrafi, F.; Coevorden, F. van; Steeghs, N.; Farag, S.; Gelderblom, H.; et al. A single digital droplet PCR assay to detect multiple KIT exon 11 mutations in tumor and plasma from patients with gastrointestinal stromal tumors. *Oncotarget* **2018**, *9*, 13870–13883.
3. Leest, P. van der; Hiddinga, B.; Miedema, A.; Aguirre Azpurua, M.L.; Rifaela, N.; Elst, A. ter; Timens, W.; Groen, H.J.M.; Kempen, L.C. van; Hiltermann, T.J.N.; et al. Circulating tumor DNA as a biomarker for monitoring early treatment responses of patients with advanced lung adenocarcinoma receiving immune checkpoint inhibitors. *Mol Oncol* **2021**,*15*, 2910–2922.
4. Lamy, P.-J.; Leest, P. van del; Lozano, N.; Becht, C.; Duboeuf, F.; Groen, H.J.M.; Hilgers, W.; Pourel, N.; Rifaela, N.; Schuuring, E.; et al. Mass Spectrometry as a Highly Sensitive Method for Specific Circulating Tumor DNA Analysis in NSCLC: A Comparison Study. *Cancers (Basel)* **2020**, *12*, 3002.
5. Gray, J.E.; Okamoto, I.; Sriuranpong, V.; Vansteenkiste, J.; Imamura, F.; Lee, J.S.; Pang, Y.-K.; Cobo, M.; Kasahara, K.; Cheng, Y.; et al. Tissue and Plasma EGFR Mutation Analysis in the FLAURA Trial: Osimertinib versus Comparator EGFR Tyrosine Kinase Inhibitor as First-Line Treatment in Patients with EGFR-Mutated Advanced Non-Small Cell Lung Cancer. *Clin Cancer Res* **2019**, *25*, 6644–6652.
6. Belloum, Y.; Janning, M.; Mohme, M.; Simon, R.; Kropidlowski, J.; Sartori, A.; Irwin, D.; Westphal, M.; Lamszus, K.; Loges, S.; et al. Discovery of Targetable Genetic Alterations in NSCLC Patients with Different Metastatic Patterns Using a MassARRAY-Based Circulating Tumor DNA Assay. *Cells* **2020**, *9*, 2337.
7. Mosko, M.J.; Nakorchevsky, A.A.; Flores, E.; Metzler, H.; Ehrich, M.; Boom, D.J. van den; Sherwood, J.L.; Nygren, A.O.H. Ultrasensitive Detection of Multiplexed Somatic Mutations Using MALDI-TOF Mass Spectrometry. *J Mol Diagnostics* **2016**, *18*, 23–31.
8. Weber, S.; Spiegl, B.; Perakis, S.O.; Ulz, C.M.; Abuja, P.M.; Kashofer, K.; Leest, P. van der; Aguirre Azpurua, M.A.; Tamminga, M.; Brudzewsky, D.; et al. Technical Evaluation of Commercial Mutation Analysis Platforms and Reference Materials for Liquid Biopsy Profiling. *Cancers (Basel)* **2020**, *12*, 1588.
9. Mehrotra, M.; Singh, R.R.; Loghavi, S.; Duose, D.Y.; Barkoh, B.A.; Behrens, C.; Patel, K.P.; Routbort, M.J.; Kopetz, S.; Broaddus, R.R.; et al. Detection of somatic mutations in cell-free DNA in plasma and correlation with overall survival in patients with solid tumors. *Oncotarget* **2018**, *9*, 10259–10271.
